# A Multifunctional Flexible Tactile Sensor Based on Resistive Effect for Simultaneous Sensing of Pressure and Temperature

**DOI:** 10.1002/advs.202307693

**Published:** 2023-12-28

**Authors:** Haodong Zhu, Hongyu Luo, Min Cai, Jizhou Song

**Affiliations:** ^1^ Department of Engineering Mechanics Soft Matter Research Center and Key Laboratory of Soft Machines and Smart Devices of Zhejiang Province Zhejiang University Hangzhou 310027 China; ^2^ Department of Rehabilitation Medicine The First Affiliated Hospital Zhejiang University Hangzhou 310003 China; ^3^ The State Key Lab of Brain‐Machine Intelligence Zhejiang University Hangzhou 310058 China

**Keywords:** electronic skin, flexible electronics, multifunctional sensing, tactile sensor

## Abstract

Flexible tactile sensors with multifunctional sensing functions have attracted much attention due to their wide applications in artificial limbs, intelligent robots, human‐machine interfaces, and health monitoring devices. Here, a multifunctional flexible tactile sensor based on resistive effect for simultaneous sensing of pressure and temperature is reported. The sensor features a simple design with patterned metal film on a soft substrate with cavities and protrusions. The decoupling of pressure and temperature sensing is achieved by the reasonable arrangement of metal layers in the patterned metal film. Systematically experimental and numerical studies are carried out to reveal the multifunctional sensing mechanism and show that the proposed sensor exhibits good linearity, fast response, high stability, good mechanical flexibility, and good microfabrication compatibility. Demonstrations of the multifunctional flexible tactile sensor to monitor touch, breathing, pulse and objects grabbing/releasing in various application scenarios involving coupled temperature/pressure stimuli illustrate its excellent capability of measuring pressure and temperature simultaneously. These results offer an effective tool for multifunctional sensing of pressure and temperature and create engineering opportunities for applications of wearable health monitoring and human‐machine interfaces.

## Graphical abstract

A multifunctional flexible tactile sensor based on resistive effect for simultaneous sensing of pressure and temperature is developed with patterned metal film on a soft substrate with cavities and protrusions. The decoupling of pressure and temperature sensing can be achieved by the distinguishable electromechanical behaviors of patterned metal films at different locations.

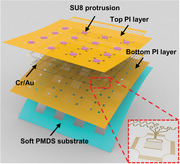

## 1 Introduction

Tactile sense (e.g., pressure, temperature, vibration, strain, etc.) is one of the important perception forms that most creatures get information from the outside world, which allows them to perform coordinated and efficient interactions with their environment.^[^ ^1^, ^2^ ^]^ The human tactile system operates through different types of tactile receptors in the skin to distinguish various mechanical and thermal stimuli. Flexible tactile sensors inspired by human skin have attracted intensive attention due to their great potential in applications of artificial limbs,^[^ ^3^, ^4^, ^5^, ^6^ ^]^ intelligent robots,^[^ ^7^, ^8^, ^9^, ^10^ ^]^ human‐machine interfaces,^[^ ^11^, ^12^, ^13^, ^14^ ^]^ and health monitoring devices.^[^ ^15^, ^16^, ^17^, ^18^ ^]^ Over the past decade, various flexible tactile sensors, based on different transduction mechanisms including resistive,^[^ ^19^, ^20^ ^]^ capacitive,^[^ ^21^, ^22^ ^]^ piezoelectric,^[^ ^23^, ^24^ ^]^ and triboelectric types,^[^ ^25^, ^26^ ^]^ with improved performances have been explored. Moreover, to enhance the reliability of signal recognition, machine learning algorithms are emerging as effective means to reveal correlations and subtle differences among multichannel datasets.^[^ ^27^, ^28^ ^]^ In order to acquire more intriguing functionalities, the trend of flexible tactile sensors is moving toward integrated flexible sensor systems, which generally refer to sensor array integration, multimodal flexible sensor integration, and integrated signal processing systems.^[^ ^29^ ^]^

Particularly, flexible tactile sensors with the ability to detect pressure and temperature simultaneously are essential for touch recognition, object manipulation, and self‐protection.^[^ ^30^, ^31^, ^32^, ^33^ ^]^ Two existing strategies have been actively adopted to measure the pressure and temperature at the same time.^[^ ^34^ ^]^ One is to combine heterogeneous sensing mechanisms by introducing temperature‐insensitive and pressure‐insensitive variables to measure pressure and temperature, respectively, including piezoresistive and thermoelectric sensors,^[^ ^35^, ^36^, ^37^ ^]^ piezoelectric/triboelectric and thermoresistive sensors,^[^ ^38^, ^39^, ^40^ ^]^ and piezo‐capacitive and thermoresistive sensors.^[^ ^41^, ^42^, ^43^, ^44^ ^]^ This strategy usually yields two different outputs, which avoid a delicate design for signal decoupling but varied materials and mechanisms in the multi‐sensing platform are often associated with high fabrication costs.^[^ ^45^ ^]^ The other is to implement a single sensing mechanism with coupled pressure and temperature variables.^[^ ^46^, ^47^, ^48^, ^49^, ^50^ ^]^ This strategy usually yields a single output (e.g., the widely used resistance), which makes it easy for large‐scale integration but needs a delicate design for signal decoupling.

Here, we report a multifunctional flexible tactile sensor based on resistive effect for simultaneous sensing of pressure and temperature. The sensor features a simple design with patterned metal film (i.e., a metal layer encapsulated by polyimides) on a soft substrate with cavities and protrusions. The decoupling of pressure and temperature sensing can be achieved by the distinguishable electromechanical behaviors of patterned metal films at different locations. The well‐established microfabrication process is used to fabricate the multifunctional flexible tactile sensor pixel and array. Systematically numerical and experimental studies have been performed to reveal the decoupled sensing mechanism. With the aid of reasonable arrangement of the metal layer in the patterned metal film, the proposed tactile sensor exhibits high performance of negligible interference, good linearity, high stability, small detection limit, and fast response. Demonstrations of the multifunctional flexible tactile sensor pixel to monitor touch, breathing, and pulse in various application scenarios involving coupled temperature/pressure stimuli, and the multifunctional flexible tactile sensor array for the perception of the spatial distribution of pressure and temperature illustrate the great potential of applications such as electronic skin and health monitoring devices.

## 2 Results and Discussion

**Figure 1:**
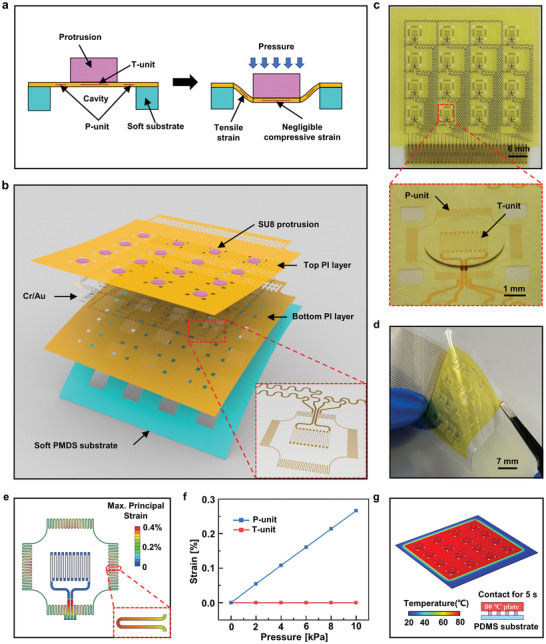
The design of the flexible tactile sensor array. a) Schematic of the pressure sensing unit (P‐unit) and temperature sensing unit (T‐unit). b) Exploded view layout of the flexible tactile sensor array. c,d) Optical images of the flexible tactile sensor array. e) The distribution of the maximum principal strain in the metal layer of the P‐unit and T‐unit calculated by FEA. f) Average strain in the metal layer of P‐unit and T‐unit as a function of applied pressure. g) Temperature profiles of the sensor array when it contacts an 80 °C hot plate for 5 s.

**Figure** **1**a shows the design and working principle of the multifunctional flexible tactile sensor. The tactile sensor consists of three key components: a protrusion (highlighted in purple), a patterned metal film (highlighted in yellow), and a soft substrate with rectangular cavities (highlighted in blue). It is known that the resistance of the patterned metal film is sensitive to both strain and temperature such that the traditional patterned metal film cannot detect the pressure and temperature simultaneously. To decouple the effect of strain and temperature on the patterned metal film, a design of relatively rigid protrusion on the patterned metal film supported by a soft substrate with cavities is proposed with abilities to define deformations in the metal layer (highlighted in red) at different locations. When the sensor touches an object, a compressive force acts on the protrusion directly, causing the patterned metal film above the cavity downward. Due to the high stiffness of the protrusion, the metal layer in the patterned metal film on the bottom of the protrusion only experiences a negligible out‐of‐plane compression‐dominated deformation under pressure such that its resistance is pressure‐insensitive. Thus, the metal layer on the bottom of the protrusion can serve as the temperature unit (T‐unit) to measure the temperature. The remaining metal layer uncovered by the protrusion experiences an appreciable in‐plane tensile deformation under pressure such that its resistance is pressure‐sensitive. Thus, the metal layer uncovered by the protrusion can serve as the pressure unit (P‐unit) to measure the pressure.

The multiple stimuli of temperature and pressure on the sensor will induce the relative resistance changes in P/T‐unit as

(1) $$\frac{\Delta R_{P}}{R_{P}^{0}}=\alpha_{\mathrm{PP}}\times \Delta P+\alpha_{TP}\times \Delta T,\;\;\frac{\Delta R_{T}}{R_{T}^{0}}=\alpha_{\mathrm{PT}}\times \Delta P+\alpha_{\mathrm{TT}}\times \Delta T$$

Here, $R_{P}^{0}$ and $R_{T}^{0}$ are the initial resistance of P‐unit and T‐unit, respectively; Δ*R*_P_ and Δ*R*_T_ are the resistance change of P‐unit and T‐unit, respectively; *α*_PP_ and *α*_PT_ are the pressure sensitivity of P‐unit and T‐unit, respectively; *α*_TP_ and *α*_TT_ are the temperature sensitivity of P‐unit and T‐unit, respectively; Δ*P* and Δ*T* are the change of pressure and temperature, respectively. The same material of P‐unit and T‐unit yields the same temperature sensitivity, i.e., *α*_TP_ = *α*_TT_. Besides, the structure design makes the pressure sensitivity of the T‐unit negligible such that Equation (1) becomes

(2) $$\frac{\Delta R_{P}}{R_{P}^{0}}=\alpha_{\mathrm{PP}}\times \Delta P+\alpha_{\mathrm{TP}}\times \Delta T,\;\frac{\Delta R_{T}}{R_{T}^{0}}\approx \alpha_{\mathrm{TT}}\times \Delta T$$

The temperature and pressure changes can then be obtained as

(3) $$\Delta T=\frac{1}{\alpha_{\mathrm{TT}}}\times \frac{\Delta R_{T}}{R_{T}^{0}},\;\Delta P=\frac{1}{\alpha_{\mathrm{PP}}}\times \left(\frac{\Delta R_{P}}{R_{P}^{0}}-\frac{\Delta R_{T}}{R_{T}^{0}}\right)$$

The combination of P‐unit and T‐unit enables the multifunctional sensing of temperature and pressure at the same time. Under the multiple stimuli of pressure and temperature, the resistance change of the T‐unit is only affected by the temperature without the interference of pressure and can reflect the accurate sensing signal of temperature. While the resistance change of the P‐unit is affected by both pressure and temperature, the removal of the resistance change of the T‐unit from that of the P‐unit will eliminate the interference of temperature and yield the accurate sensing signal of pressure.

To demonstrate the concept of the multifunctional sensor with the ability to measure the pressure and temperature simultaneously, we designed a flexible tactile sensor array (40.4 mm × 42.82 mm) consisting of 4 × 4 sensing pixels (4 mm × 4 mm) with more design details illustrated in Figure S1 (Supporting Information). Figure 1b shows the exploded view layout of the flexible tactile sensor array. The bottom blue layer is a soft polydimethylsiloxane (PDMS) substrate (150 µm in thickness) with 16 rectangular cavities (4 mm × 4 mm × 150 µm). The top discrete purple cylinders represent the protrusion array (SU‐8 photoresist; 2.5 mm in diameter and 150 µm in height). The gray metal layer sandwiched between two yellow polyimide (PI) encapsulating layers (2.5 µm in thickness) yields the patterned metal film. The metal layer is made of Cr/Au with a thickness of 5/40 nm and a width of 20 µm for the P‐unit and T‐unit, and a thickness of 5/160 nm and a width of 80 µm for the connecting wire. The different treatments on the dimension of the P/T‐units and the connecting wire significantly reduce the effect of the connecting wire on the resistance change of P/T‐units. To improve the pressure sensing performance of the P‐unit, four rectangular via holes (0.75 mm × 0.75 mm × 2.5 µm) are introduced in the patterned metal film of the sensor pixel, which breaks the seal of the cavities and thus enhances the strain of the P‐unit. It should be noted that the object applying pressure should be larger than the distance between the protrusions. If the object is smaller than the distance between the protrusions, the pressure sensor will not work. Figure 1c shows an optical image of the multifunctional flexible tactile sensor array prepared by the well‐established microfabrication processes (Figure S2, Supporting Information) with key fabrication details described in the Experimental Section. To avoid cross‐talk in the sensor array, one end of each temperature and pressure sensor is connected to a common connection wire, i.e., the potential at this end of each sensor is the same. The insert of Figure 1c shows the enlarged view of a single sensor pixel consisting of one P‐unit and one T‐unit. The multifunctional flexible tactile sensor array exhibits good flexibility (Figure 1d) and can be easily attached to surfaces of different shapes.

In order to further validate the multifunctional sensing function of the proposed flexible tactile sensor, finite element analysis (FEA), with details described in the Experimental Section, was carried out to investigate the strain levels in the metal layers of P/T‐unit under pressure and temperature loadings. It should be noted that the strain level in the metal layer determines the resistance change, reflecting the sensing performance of the sensor. The higher the strain level, the better the sensing performance. Figure 1e shows the distribution of the maximum principal strain of the metal layer in the sensor pixel subjected to a uniform pressure of 10 kPa. It is shown that the strain level of the metal layer in the P‐unit is much larger than that in the T‐unit. To quantitatively compare the strain levels of the metal layer in P/T‐unit, the average strains as functions of the applied pressure are shown in Figure 1f. The huge difference in the slope, reflecting the sensor sensitivity of the pressure sensing, indicates that the T‐unit is pressure‐insensitive with a negligible small slope compared to that of the pressure‐sensitive P‐unit. In some cases, the object may contact the sensor at an angle with horizontal and vertical pressures. FEA was carried out to investigate the influence of horizontal pressure on the sensor (Figure S3, Supporting Information), which indicates that our sensor is only good for cases subjected to vertical pressure. Figure 1g shows the effect of thermal load on the sensor. The temperature of the P‐unit is almost the same as that of the T‐unit when the sensor array contacts an 80 °C hot plate for 5 s, which indicates that the temperature effect on the P‐unit could be easily eliminated by the measurement of the T‐unit. These FEA results clearly show that the proposed design provides an effective thermomechanical decoupling mechanism for measuring pressure and temperature simultaneously.

**Figure 2:**
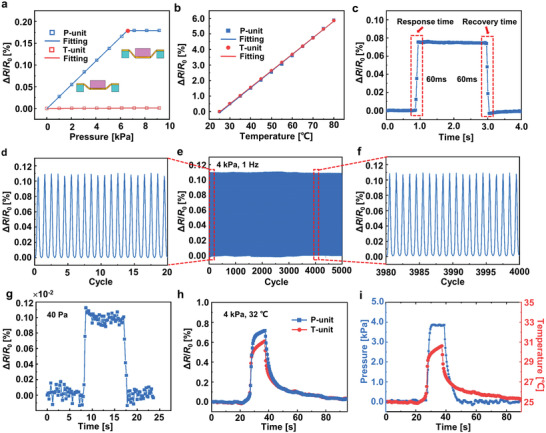
Characterization of the multifunctional sensing performance of the flexible tactile sensor. a) Pressure‐response (Δ*R*/*R*_0_) curves of the sensor. b) Temperature‐response (Δ*R*/*R*_0_) curves of the sensor. c) Dynamic response of the P‐unit under a pressure of 3 kPa. d–f) Cyclic loading on the sensor for 5000 times at a frequency of 1 Hz under a pressure of 4 kPa. g) The pressure response under a small magnitude of pressure (40 Pa). h) Real‐time recorded resistance change rates of P/T‐unit subjected to a pressure of 4 kPa and a temperature of 32 °C simultaneously. i) The decoupled pressure and temperature measured by the sensor.

A series of static and dynamic experiments were carried out to characterize the multifunctional sensing performance of the flexible tactile sensor. We applied a gradually increasing displacement to the sensor by using a universal material testing machine and simultaneously recorded the magnitude of the applied pressure and the resistance of the P/T‐unit. **Figure** **2**a shows the relationship between the resistance change rate (Δ*R*/*R*_0_ with Δ*R* as the measured resistance change and *R*_0_ as the measured initial resistance without loading) and the applied pressure. Again, the T‐unit response is much smaller than that of the P‐unit, which indicates that the T‐unit is press‐insensitive. It is observed that the response of the P‐unit has two distinct regions of linear response region and constant response region separated by a critical applied pressure (6.1 kPa for this case), which depends on the cavity dimension. When the applied pressure is below 6.1 kPa, the P‐unit has a good linearity (*R* ^2^ = 0.998) with a sensitivity (*S* = (Δ*R*/*R*_0_)/*F*) of 2.71 × 10^−4^ kPa^−1^. When the pressure is greater than 6.1 kPa, the resistance change rate of the P‐unit remains almost unchanged due to the touch of the protrusion with the bottom of the cavity. Further increasing the pressure will have no influence on the in‐plane tensile strain of the metal layer in the P‐unit. This unique feature defines the maximum pressure sensing range of the tactile sensor and also provides a protection mechanism for the sensor. Different from the P‐unit, the resistance change rate of the T‐unit increases monotonically and linearly with the applied pressure with a small slope of 1.52 × 10^−6^ kPa^−1^. The pressure sensing sensitivity (i.e., slop) of the P‐unit is 178 times larger than that of the T‐unit such that the effect of pressure on the T‐unit is negligible and the T‐unit is pressure‐insensitive. The temperature calibration was carried out by water bath on a hot plate. The liquid environment is created by boiled DI water in order to exclude the influence or disturbing of the air bubble (generated while heating) on the temperature and resistance measurement.^[^ ^51^ ^]^ Figure 2b shows the relationship between the resistance change rate and the applied temperature. As expected, the P‐unit and T‐unit share the same temperature sensing response with good linearity (*R* ^2^ = 0.999, slope: 1.04 × 10^−3^ °°C^−1^).

In order to study the dynamic response of the P‐unit, fast loading/unloading of the sensor was carried out. The resistance change rate of the P‐unit as a function of time under a pressure of 3 kPa is recorded in Figure 2c with a sampling frequency of 50 Hz. The response time and recovery time are both on the order of 60 ms, which indicates that the sensor is good for a dynamic loading environment. To evaluate the stability and robustness of the P‐unit, a fatigue testing system was used to apply 4 kPa pressure at a frequency of 1 Hz for a total of 5000 times. Figure 2e records the resistance change rate of the P‐unit during the fatigue test. It can be observed that the performance of the P‐unit has no obvious change after 5000 times of loading/unloading cycling test. The responses of 0–20 times of loading/unloading and 3980–4000 times of loading/unloading are shown in Figure 2d,f, respectively. These fatigue test results show that the sensor has a high stability to measure the pressure. Figure 2g shows the ability of the sensor to detect small pressures. When a small piece of silicone, corresponding to a small applied pressure of 40 Pa, is placed on and removed from the sensor, the resistance change rate of the P‐unit is ≈0.0012%, which indicates that the minimum pressure detection limit of the sensor may be lower than 40 Pa. The performance of our device was compared with other sensors that focus on the decoupling of the pressure and temperature (see Table S1, Supporting Information). Our pressure sensor exhibits good linearity, a small detection limit, and a fast response.

To evaluate the multifunctional sensing performance of the sensor, a film heater was integrated into the probe of the universal materials testing machine to simultaneously apply a pressure load of 4 kPa and a temperature load of 32 °C to the sensor. The resistance change rates of P‐unit and T‐unit are shown in Figure 2h. The response of the T‐unit is only affected by temperature, while that of the P‐unit is affected by both pressure and temperature. The pressure signal can be obtained by subtracting the response of the T‐unit from that of the P‐unit. Figure 2i shows the measured pressure and the temperature after decoupling the signals of P/T units. Before the film heater contacts the sensor, as expected, the pressure is zero and the temperature is the same as the ambient temperature (25 °C). After the film heater contacts the sensor at 27 s, the pressure and temperature start to rise at the same time. The pressure becomes stable within 1 s and yields a value of the applied pressure (4 kPa) while the temperature continues to rise since it needs a much longer time to reach the stable state. When the film heater leaves the sensor, the pressure begins to decline to zero quickly, while the temperature recovers slowly to the ambient temperature. It should be noted that the response time (≈1 s) and recovery time (≈50 s) of the temperature sensor are much longer than those of the pressure sensor. These results show that the sensor has the excellent capability of multifunctional sensing of temperature and pressure.

**Figure 3:**
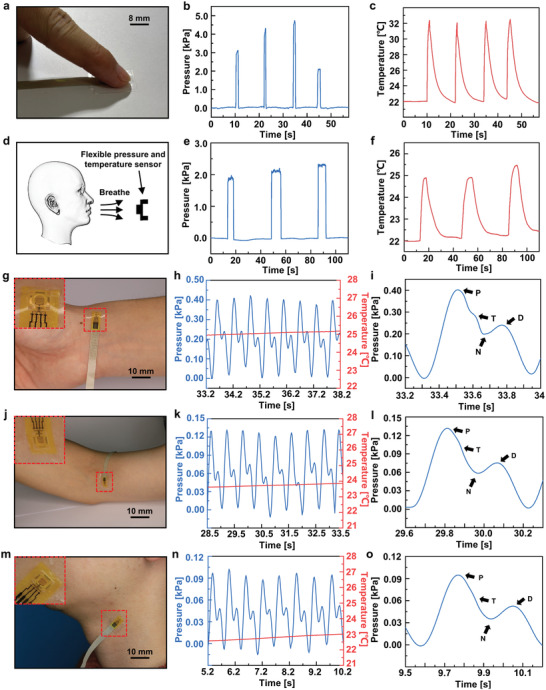
Demonstrations of the flexible tactile sensor in applications involving both pressure and temperature variations. a) Optical image of a fingertip pressing the sensor. b) Pressure and c) temperature responses of the sensor for four arbitrary fingertip presses. d) Schematic of noncontact detection of breathing. e) Pressure and f) temperature responses of the sensor for three respirations. g) Optical image for radial pulse wave monitoring. h) Real‐time radial artery pressure. i) Representative pulse waveform of the radial artery extracted from (h). j) Optical image for brachial pulse wave monitoring. k) Real‐time brachial artery pressure. l) Representative pulse waveform of the brachial artery extracted from (k). m) Optical image for carotid pulse wave monitoring. n) Real‐time carotid artery pressure. o) Representative pulse waveform of the carotid artery extracted from (n).

The developed flexible tactile sensor can be applied in various application scenarios involving both pressure and temperature variations. **Figure** **3**a shows the optical image of a sensor pixel touched by a fingertip. The press of the fingertip induces pressure on the sensor and at the same time, the human body temperature induces a temperature change on the sensor. The measured pressure and temperature are shown in Figure 3b,c, respectively (see Figure S4, Supporting Information for the real‐time recorded resistance change rates of P/T‐unit), under different fingertip presses. The measured pressures are 3.11, 4.32, 4.73, and 2.10 kPa, respectively, and the temperatures are all ≈32 °C for the four arbitrary fingertip presses. The measured temperature by the sensor is consistent with that measured by the infrared thermal image (see Figure S5, Supporting Information). When the sensor was pressed by a cotton swab with a temperature close to room temperature (≈24 °C), the measured temperature remained at room temperature, and only the pressure response changed accordingly (see Figure S6, Supporting Information). In addition to measuring contact signals, the proposed sensor can also measure noncontact signals such as airflow during human breathing as illustrated in Figure 3d. It should be noted that human breathing induces both pressure and temperature changes simultaneously. The sensor is placed ≈10 cm in front of the mouth. Figure 3e,f the measured pressure and temperature, respectively, under three respirations (see Figure S7, Supporting Information for the real‐time recorded resistance change rates of P/T‐unit). These results illustrate the promising potential of the proposed sensor for diagnosing respiratory diseases.

The pulse wave is an important health indicator of the human body.^[^ ^52^, ^53^ ^]^ Human arteries (carotid artery, brachial artery, radial artery, etc.) are usually buried deep end such that pulse wave detected from the skin surface is affected by muscle, fat, and other skin issues.^[^ ^54^, ^55^, ^56^ ^]^ Moreover, skin temperature affects the contraction and relaxation of blood vessels through the changes in arterial stiffness and radial pressure.^[^ ^57^, ^58^ ^]^ Thus, a flexible tactile sensor that can monitor the pulse pressure and temperature simultaneously is highly desired and helpful to achieve an accurate pulse signal. The good mechanical flexibility, high sensitivity, and good stability of our flexible tactile sensor can serve as a potential wearable device to monitor the pulse wave. Flexible tactile sensors are integrated onto the skin surface by using stretchable acrylic tape to measure pulse waves in various arteries, including the radial artery (Figure 3g), brachial artery (Figure 3j), and carotid artery (Figure 3m). For each artery, the pressure and the temperature were measured for 50 s from the time the sensor was attached to the skin surface. Figure 3h shows the radial pulse wave (see Figure S8, Supporting Information for the complete monitoring period of 50 s) for the 5 s period with a representative pulse waveform illustrated in Figure 3i. Figure 3k shows the brachial pulse wave (see Figure S9, Supporting Information for the complete monitoring period of 50 s) for the 5 s period with a representative pule waveform illustrated in Figure 3l. Figure 3n shows the carotid pulse wave (see Figure S10, Supporting Information for the complete monitoring period of 50 s) for the 5 s period with a representative pule waveform illustrated in Figure 3o. It is observed that typical characteristic peaks including the percussion wave (P wave), tidal wave (T wave), diastolic wave (D wave), and valley (N) are all captured in the radial, brachial, and carotid pulse waves although the T‐wave is not obvious in brachial and carotid pulse waves. These results illustrate the promising potential of the proposed sensor for pulse diagnosis of cardiovascular diseases.

**Figure 4:**
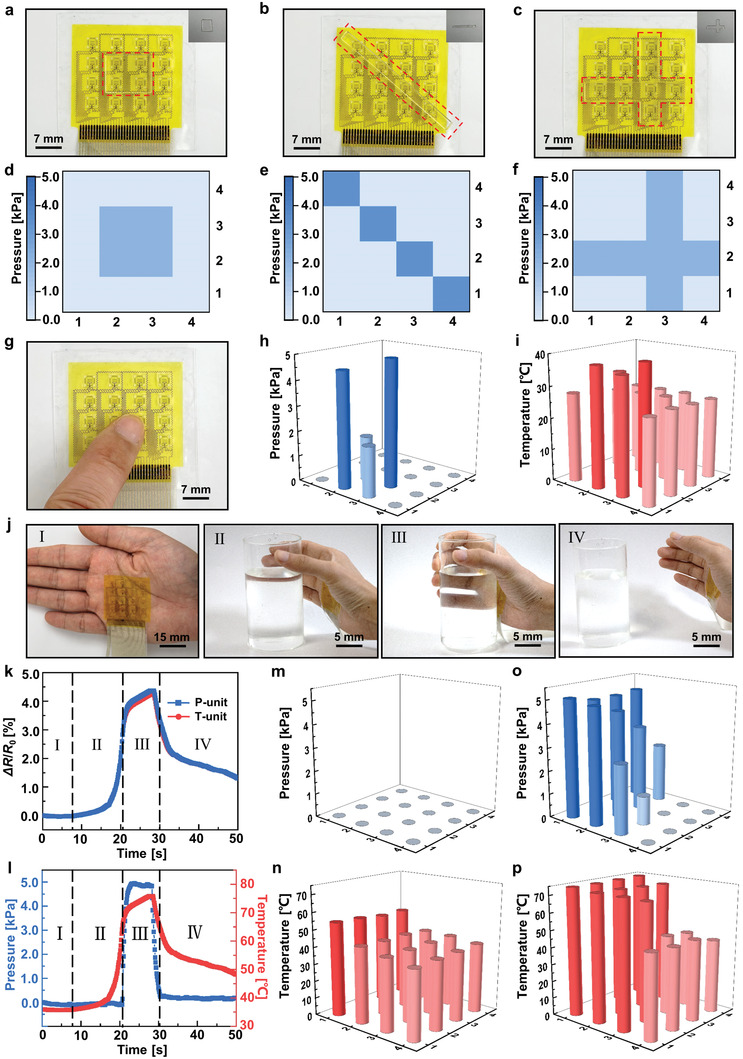
Flexible tactile sensor array for spatial sensing of pressure and temperature. a–c) Optical images of the rectangular, stick‐shaped, and cross‐shaped acrylic plates placed on the sensor array. d–f) The corresponding pressure distributions are measured by the sensor array. g) Optical image of a finger pressing on the sensor array. h) Pressure distribution and i) temperature distribution measured by the sensor array. j) Optical image of the process of grabbing/releasing a cup of hot water with a sensor array mounted on the palm: I) far away from the cup, II) approaching the cup, III) grabbing the cup, and IV) releasing the cup. k) Real‐time recorded resistance change rates of P/T‐unit at position (1,2). l) The correspondingly measured pressure and temperature. m) Pressure distribution and n) temperature distribution at 19.9 s in stage II. o) Pressure distribution and p) temperature distribution at 25.6 s in stage III.

The flexible tactile sensor array can be used for spatial sensing of pressure and temperature. Rectangular (**Figure** **4**a), stick‐shaped (Figure 4b), and cross‐shaped (Figure 4c) acrylic plates are placed on the sensor array with the pressure distributions measured and shown in Figure 4d–f respectively. The sensor array can easily identify the shape and weight distributions of the target object. Figure 4g shows the optical image of a fingertip pressing on the sensor array. The fingertip touches four sensing pixels and leaves all the other sensing pixels untouched. The measured pressures for the touched sensing pixels are 4.57, 1.96, 1.76, and 4.99 kPa, respectively. Figure 4h reconstructs the pressure distribution via the 3D bar chart. The pressures have noticeably different values due to the protuberant shape of the fingertip. The measured temperatures for the touched sensing pixels are 36.9, 35.4, 34.5, and 37.7 °C, respectively, with no obvious differences in magnitudes. Figure 4i shows the temperature distribution with untouched surrounding sensors yielding the room temperature of 25 °C.

The flexible tactile sensor array can also be integrated into the skin (or glove) surface for spatial sensing of pressure and temperature due to its good mechanical flexibility. Figure 4j‐I shows the optical image of the sensor array attached to a palm by using stretchable acrylic tape. The attached sensor array is adopted to study the process of grabbing/releasing a cup of hot water, which involves both pressure and temperature changes. As the palm approaches (Figure 4j‐II), grabs (Figure 4j‐III), and releases (Figure 4j‐IV) the cup of hot water, the resistance change rates of P/T units are recorded with the values of sensor pixel at the position (1,2) shown in Figure 4k. The measured pressure and temperature responses are shown in Figure 4l. The whole grabbing/releasing process can be divided into four stages. In the first stage, when the palm is far away from the cup, the pressure is zero and the temperature remains the same as the body surface temperature. In the second stage, when the palm is close to the water cup, the pressure is still zero since the sensor array (i.e., the palm) doesn't contact the cup while the temperature starts to rise gradually due to the thermal radiation of hot water. In the third stage, when the palm grabs the cup, the contact between the palm and the cup induces a pressure response and the temperature continues to increase due to the thermal conduction of hot water to the sensor array. In the fourth stage, when the palm releases the cup and leaves, the pressure drops rapidly to zero and the temperature gradually decreases to the body temperature. Figure 4m,n shows the pressure and temperature distributions of the sensor array at 19.9 s, respectively, which correspond to a state in the second stage. Figure 4o,p show the pressure and temperature distributions of the sensor array at 25.6 s, respectively, which correspond to a state in the third stage. These results illustrate the promising potential of the proposed sensor array for intelligent skin of robots with multifunctional tactile sensing capabilities in human‐machine interfaces.

## 3 Conclusion

Here, we report a multifunctional flexible tactile sensor, which features a simple design with patterned metal film on a soft substrate with cavities and protrusions, based on resistive effect for simultaneous sensing of pressure and temperature. Experimental and numerical studies show that the proposed sensor design provides a novel decoupling mechanism of pressure and temperature sensing via the reasonable arrangement of metal layers in the patterned metal film. The multifunctional flexible tactile sensor exhibits high performance including good linearity (*R* ^2^ = 0.998), high stability (>5000 loading‐unloading cycles), small detection limit (<40 Pa), fast response (60 ms), good mechanical flexibility, and good microfabrication compatibility, and so on. A series of experiments were carried out to illustrate the multifunctional sensing capability of the tactile sensor, including the detection of pressure and temperature during the touch, breathing, and artery pulsing. Demonstration of the multifunctional flexible tactile sensor array in grabbing/releasing objects for the perception of the spatial distribution of pressure and temperature illustrates the great potential of electronic skin and health monitoring devices. These results provide an important scientific foundation for constructing the multifunctional tactile sensor by implementing a single sensing mechanism. Despite the high performance of the proposed sensor, it still has some limitations such as complex fabrications and limited working range. Future efforts could focus on overcoming these limitations as well as integrating the sensor array with the readout circuit and energy supply to push it to practical applications.

## 4 Experimental Section

### Fabrication of the Flexible Tactile Sensor Array

To prepare the patterned metal film, the PI precursor solution (ZKPI‐305IIE, POME) was first spin‐coated on a cleaned glass substrate at 4000 rpm for 60 s, followed by curing at 85 °C for 30 min, 110 °C for 60 min, and 230 °C for 150 min, to yield the PI layer. The Cr/Au (5/50 nm) metal layer of the P/T‐unit was patterned on the PI through photolithography and electron beam evaporation (DZS‐500, SKY). Next, another Cr/Au (5/160 nm) metal layer was deposited on the PI layer by the same process to form the connecting wire. Another PI layer as encapsulation was spin‐coated (4000 rpm for 60 s, 2.5 µm) on it and patterned by an inductively coupled plasma etching (ICP‐100A, TAILONG). The SU‐8 (GM1070, Gersteltec) was spin‐coated (650 rpm for 40 s, 150 µm) on it and patterned photolithography to form protrusions. The sensor array was then released in the buffer oxidation etching (BOE) solution (RESEMI) and picked up by a water‐soluble tape (ASW‐1, AQUASOL). Then, a thin layer of Cr/SiO2 (5/30 nm) was deposited on the bottom PI surface by electron beam evaporation. To prepare the PDMS substrate, the poly(styrene sulfonic acid) solution (MACKLIN) was first spin‐coated on a cleaned glass substrate at 2500 rpm for 30 s, followed by curing at 100 °C for 5 min. A PDMS resin and curing agent were mixed at a ratio of 10:1 to form a PDMS solution, and it was degassed in a vacuum oven for 20 min. Then, the PDMS solution was spin‐coated on the poly(styrene sulfonic acid) layer at 500 rpm for 40 s, to form a PDMS layer of 150 µm. The cavities of PDMS were cut by the laser etching system (VLS2.30 DT ‐SYS, UNIVERSAL). The sensor array was transferred onto the PDMS substrate with its surface activated by ozone. Last, the water‐soluble tape and the poly(styrene sulfonic acid) layer were dissolved in water, and a flexible anisotropic conductive film (ACF) cable was thermally (150 °C for 30 s) bonded to the contact pads of the flexible tactile sensor array at one side and to the external data acquisition circuit at the other side.

### Mechanical Analysis of the Tactile Sensor

To investigate the strain distributions of the P‐unit and the T‐unit subjected to pressure, three‐dimensional (3D) finite element models were established in ABAQUS. The thicknesses of PDMS, chromium, gold, and PI were 150 µm, 5 nm, 40 nm, and 5 µm, respectively. The chromium layer is negligible because it is extremely thin. The elastic moduli of PDMS, PI, gold, and SU‐8 were 1.5 MPa, 2.5 GPa, 78.5 GPa, and 4.6 GPa, respectively. The Poisson's ratios of PDMS, PI, gold, and SU‐8 were 0.48, 0.34, 0.42, and 0.25, respectively. A pressure of 10 kPa was applied to the P/T‐unit.

### Thermal Analysis of the Tactile Sensor

To investigate the temperature distributions of the P‐unit and the T‐unit, 3D finite element models were established in COMSOL. The thermal conductivities of PDMS, PI, and SU‐8 were 0.16, 0.3, and 0.2 W m^−1^ K^−1^, respectively. The heat capacities of PDMS, PI, and SU‐8 were 1460, 1090, and 550 J kg^−1^ K^−1^, respectively. The densities of PDMS, PI, and SU‐8 were 970, 1320, and 3100 kg m^−3^, respectively. The Cr/Au layer is negligible because it is extremely thin. The surface of the tactile sensor maintained a constant temperature of 80 °C for 5 s.

### Electrical Measurement of the Sensing Performance of the Sensor

The flexible tactile sensor array was connected to the electrical signal recording equipment by a flexible ACF cable. The electrical resistance of the P/T‐unit was recorded by a digital multimeter (DAQ6510, Keithley) and a 40‐channel differential multiplexer module (7708).

### Fatigue Test of the Sensor

Fatigue tests were carried out by a fatigue testing system (M‐100, CARE Measurement & Control) with a loading pillar (45 mm in diameter). The compression force was selected as 19.6 mN. The fatigue cycling was carried out under the sinusoidal force control at a loading frequency of 1 Hz and an amplitude of 19.6 mN. Meanwhile, the flexible tactile sensor was connected to the digital multimeter for electrical measurement.

## Conflict of Interest

The authors declare no conflict of interest.

## Supporting information

Supporting Information

## Data Availability

The data that support the findings of this study are available from the corresponding author upon reasonable request.
