# Supplementary material for: A Multifunctional Flexible Tactile Sensor Based on Resistive Effect for Simultaneous Sensing of Pressure and Temperature
Source: Adv Sci (Weinh). 2023 Dec 28;11(6):2307693. doi: 10.1002/advs.202307693 (PMC10853712; doi:10.1002/advs.202307693)
Supplement: Supplementary file 1 — Supporting Information [file ADVS-11-2307693-s001.pdf]

ADVANCED SCIENCE
Open Access

Supporting Information

for *Adv. Sci.*, DOI 10.1002/advs.202307693

A Multifunctional Flexible Tactile Sensor Based on Resistive Effect for Simultaneous Sensing of Pressure and Temperature

*Haodong Zhu, Hongyu Luo, Min Cai and Jizhou Song**

Supporting Information

A Multifunctional Flexible Tactile Sensor Based on Resistive Effect for Simultaneous Sensing of Pressure and Temperature

Haodong Zhu[1], *Hongyu Luo*[1], *Min Cai*[1], *and Jizhou Song*[1,2,3]*

[1]Department of Engineering Mechanics, Soft Matter Research Center, and Key Laboratory of Soft Machines and Smart Devices of Zhejiang Province, Zhejiang University, Hangzhou 310027, China

[2]Department of Rehabilitation Medicine, The First Affiliated Hospital, Zhejiang University, Hangzhou 310003, China

[3]The State Key Lab of Brain-Machine Intelligence, Zhejiang University, Hangzhou 310058, China

**To whom correspondence should be addressed jzsong@zju.edu.cn*

This PDF file includes:

Figs. S1 to S10
Table S1

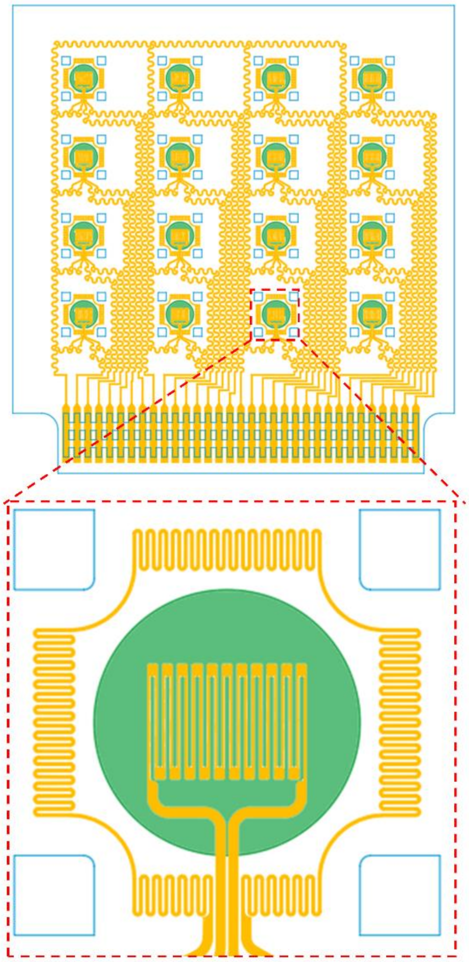

Figure S1. Structural design of the flexible tactile sensor array.

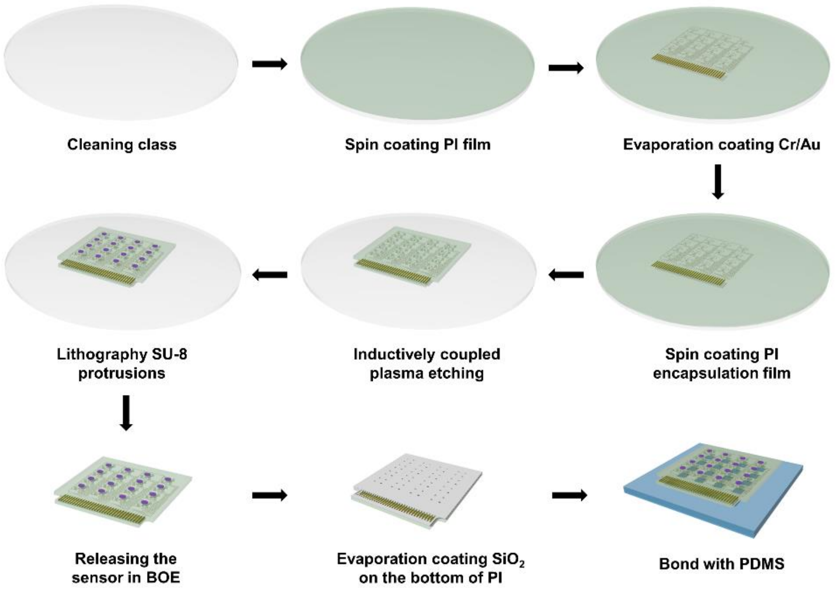

Figure S2. Schematic fabrication process of the flexible tactile sensor array.

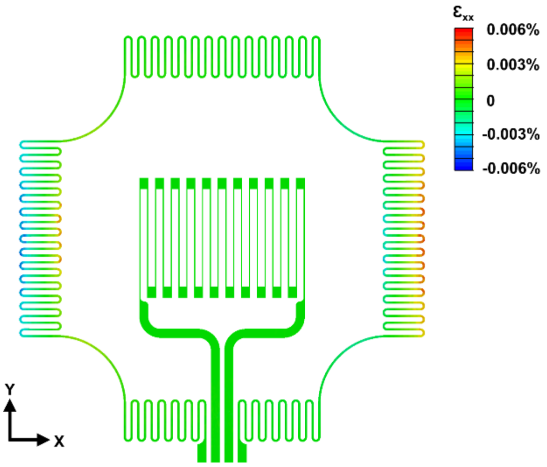

Figure S3. The distribution of ε_{xx} in the metal layer of P-unit and T-unit calculated by FEA under a horizontal pressure of 0.2 kPa.

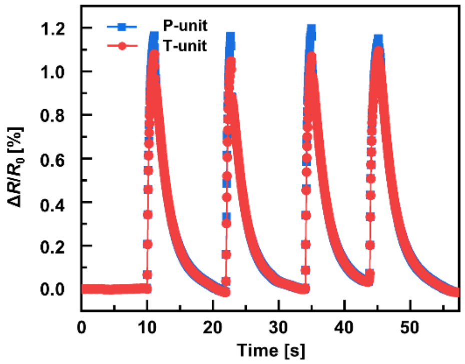

Figure S4. Real-time recorded resistance change rates of P/T-unit subjected to four arbitrary fingertip presses.

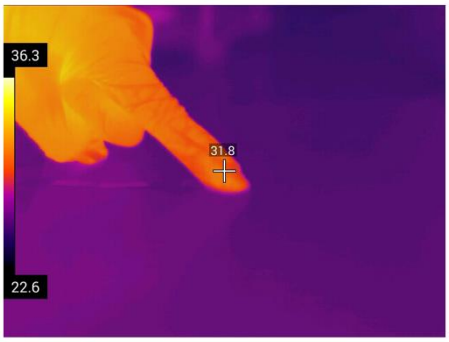

Figure S5. Infrared thermal image of the flexible tactile sensor pressed by the fingertip.

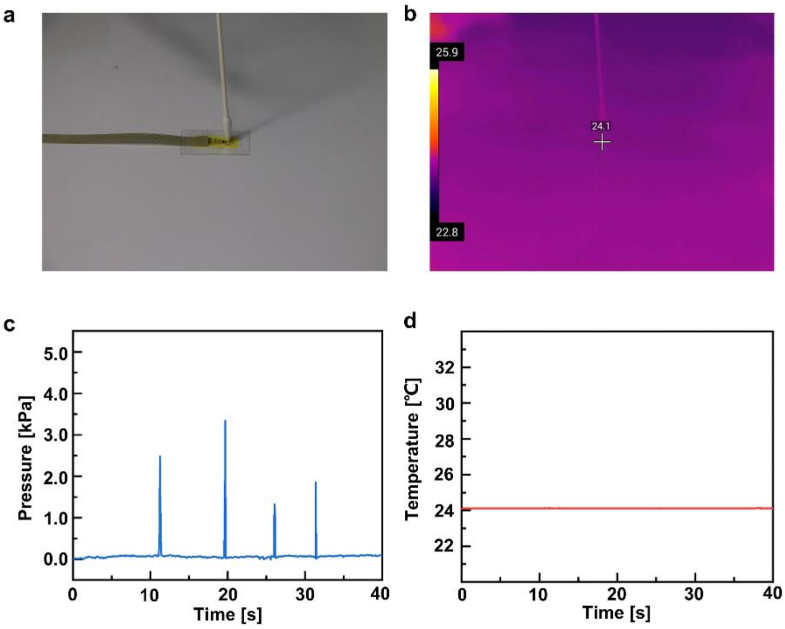

Figure S6. The flexible tactile senor touched by a cotton swab. (a) Optical image, (b) Infrared thermal image, and (c-d) the correspondingly measured pressure and temperature.

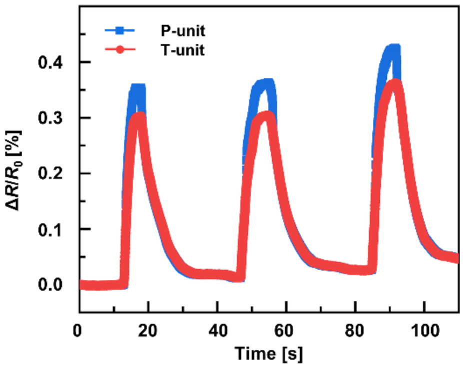

Figure S7. Real-time recorded resistance change rates of P/T-unit for three respirations.

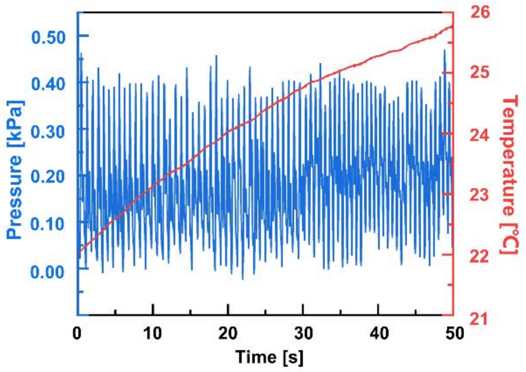

Figure S8. Real-time measured pressure and temperature of radial artery.

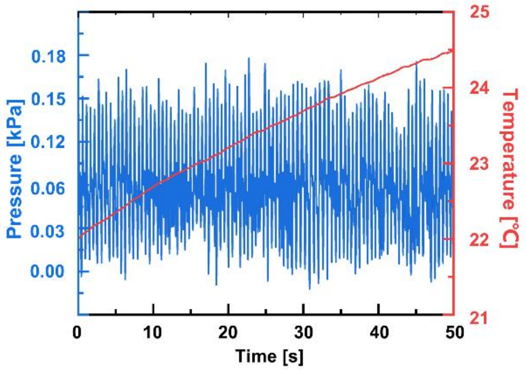

Figure S9. Real-time measured pressure and temperature of brachial artery.

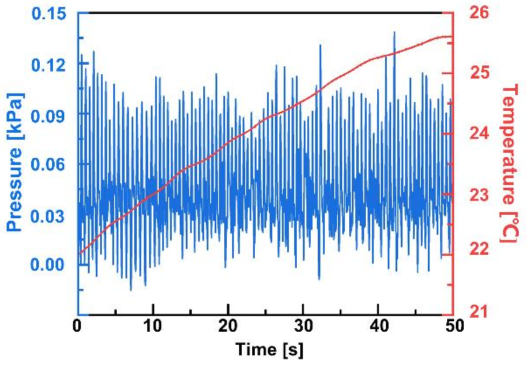

Figure S10. Real-time measured pressure and temperature of carotid artery.

Table S1. Comparison of our device with other sensors that focus on the decoupling of the pressure and temperature.

Ref.	Type of pressure sensor	Sensitivity of pressure [kPa^{-1}]	Linear range [kPa]	Response time [ms]	Recovery time [ms]	Minimum detection limit [Pa]	Type of temp. sensor	Sensitivity of temp. [K^{-1}]
**This work**	Piezoresistive	2.71e-4	0-6.1	60	60	40	Thermoresistive	1.04e-3
**37**	Piezoresistive	28.9	-	20	20	100	Thermoelectric	35.5 μV
**40**	Triboelectric	5.07 V	0.2-1.72	-	-	200	Thermoresistive	0.015
**42**	Capacitive	0.75 (0-2) 0.0549 (2-50)	0-2, 2-50	94	134	2	Thermoresistive	2.84e-3
**44**	Capacitive	3.15e-3	0-150	70	1350	-	Thermoresistive	1.5e-3
**46**	Piezoresistive	1185.8 (0-5), 50.4 (5-40), 17.3 (40-80)	0-5, 5-40, 40-80	23	16	2.4	Thermoresistive	-
**47**	Piezoresistive	2	0-0.25	20	20	3	Thermoresistive	3.2e-3
**48**	Piezoelectric	0.044 V	15.4-27.6	**-**	-	-	Pyroelectric	0.048 V
